# Predictive value of serum procalcitonin for both initial and repeated immunoglobulin resistance in Kawasaki disease: a prospective cohort study

**DOI:** 10.1186/s12969-019-0379-5

**Published:** 2019-11-27

**Authors:** Shuran Shao, Chunyan Luo, Kaiyu Zhou, Yimin Hua, Mei Wu, Lei Liu, Xiaoliang Liu, Chuan Wang

**Affiliations:** 1 Department of Pediatric Cardiology, West China Second University Hospital, Sichuan University, No. 20, Section 3, South Renmin Road, Chengdu, 610041 Sichuan China; 2 West China Medical School of Sichuan University, Chengdu, Sichuan China; 3 Department of Radiology, West China Hospital, Sichuan University, Chengdu, Sichuan China; 4 The Cardiac development and early intervention unit, West China Institute of Women and Children’s Health, West China Second University Hospital, Sichuan University, Chengdu, Sichuan China; 5 Key Laboratory of Birth Defects and Related Diseases of Women and Children (Sichuan University), Ministry of Education Chengdu, Chengdu, Sichuan China; 6 Key Laboratory of Development and Diseases of Women and Children of Sichuan Province, West China Second University Hospital, Sichuan University, Chengdu, Sichuan China

**Keywords:** Procalcitonin, Kawasaki disease, Prediction, Immunoglobulin resistance

## Abstract

**Background:**

Intravenous immunoglobulin (IVIG) resistance prediction is one pivotal topic of interests in Kawasaki disease (KD) since those patients with KD resistant to IVIG might improve of an early-intensified therapy. Data regarding predictive value of procalcitonin (PCT) for IVIG resistance, particularly for repeated IVIG resistance in KD was limited. This study aimed to testify the predictive validity of PCT for both initial and repeated IVIG resistance in KD.

**Methods:**

A total of 530 KD patients were prospectively recruited between January 2015 and March 2019. The clinical and laboratory data were compared between IVIG-responsive and IVIG-resistant groups. Multivariate logistic regression analysis was applied to determine the association between PCT and IVIG resistance. Receiver operating characteristic (ROC) curves analysis was further performed to assess the validity of PCT in predicting both initial and repeated IVIG resistance.

**Results:**

The serum PCT level was significantly higher in initial IVIG-resistance group compared with IVIG-response group (*p* = 0.009), as well as between repeated IVIG responders and nonresponders (*p* = 0.017). The best PCT cutoff value for initial and repeated IVIG resistance prediction was 1.48 ng/ml and 2.88 ng/ml, respectively. The corresponding sensitivity was 53.9 and 51.4%, while the specificity were 71.8 and 73.2%, respectively. Multivariate logistic regression analysis failed to identify serum PCT level as an independent predictive factor for both initial and repeated IVIG resistance in KD.

**Conclusions:**

Serum PCT levels were significantly higher in IVIG nonresponders, but PCT may not be suitable as a single marker to accurately predict both initial and repeated IVIG resistance in KD.

## Background

Kawasaki Disease (KD) is an acute general vasculitis of unknown etiology that mainly occurring in infants and children younger than 5 years of age [1]. While timely initiation of treatment with high-dose intravenous immunoglobulin (IVIG) can effectively reduce the development of coronary artery lesions (CALs), approximately 10%~ 20% patients do not respond to IVIG treatment, and have a higher risk of CALs [2, 3]. For children with initial IVIG resistance, repeated IVIG infusion (2 g/Kg given as a single intravenous infusion) is recommended by many experts despite there are currently no robust data from clinical trails to guide the clinician in the choice of therapeutic agents [4–6]. However, approximately 10% of patients are refractory to both the initial and repeated IVIG therapy [7], and often require additional interventions, such as corticosteroid [8, 9], infliximab [10, 11], plasma exchange [12, 13] and cytotoxic agents [14, 15]. Therefore, early identification of both the initial and repeated IVIG resistance is of amount importance to reduce CALs, and most importantly, to lower the medical costs. This is particularly important in patient population outside of Japan since risk-scoring systems developed in Japan [16–18] have not been reproducible in Chinese populations, and attempts to develop similar algorithms [19–22] have been unsuccessful.

Procalcitonin (PCT), a 116-aminoacid peptide and one of the calcitonin precursors, has been widely proved to be a significant biomarker in severe bacterial infection and sepsis [23]. Various pro-inflammatory stimuli, such as interlukin-1 (IL-1), IL-2, IL-6 and tumor necrosis factor (TNF)-α, could induce the production of PCT, suggesting PCT might be correlated with an immune response [24]. Although the etiology of KD remains unknown, it is believed that the condition might result from an exaggerated immune response to an infection in patients with genetic susceptibility. Indeed, the serum PCT level was found to elevate compared to healthy controls, and appeared to be useful in differentiating KD from viral infections or autoimmune disease [25]. However, the current data regarding PCT as a predictive marker for IVIG resistance in KD patients is limited [26–28]. Most importantly, these studies were limited by small sample size and retrospective nature. Furthermore, data on the validity of serum PCT in repeated IVIG response prediction is lacking. Herein, we performed a prospective study in an appropriately large sample to assess the effectiveness of serum PCT level in identifying KD patients at risk for both initial and repeated IVIG resistance.

## Methods and materials

We prospectively recruited patients with KD who were hospitalized at the West China Second University Hospital of Sichuan University, which is the largest medical center for children in Southwest China, between January 2015 and March 2019. The diagnosis of complete and incomplete KD relies on standards recommended by AHA scientific statement for diagnosis, treatment, and long-term management of KD [1] and were confirmed by 2 experienced pediatricians (at least one of them is a KD specialist). Structured questionnaires with pre-coded questions including basic demographic information, clinical manifestations, hematological examination results, treatment details and follow up outcomes, were used for data collection. All questionnaires were pretested and revised accordingly. Two well-trained doctors conducted data collection. The questionnaires were double-checked to assure its completeness.

Informed written consent was obtained from the parents after the nature of this study had been fully explained to them. The University Ethics Committee on Human Subjects at Sichuan University approved the study.

**Fig. 1:**
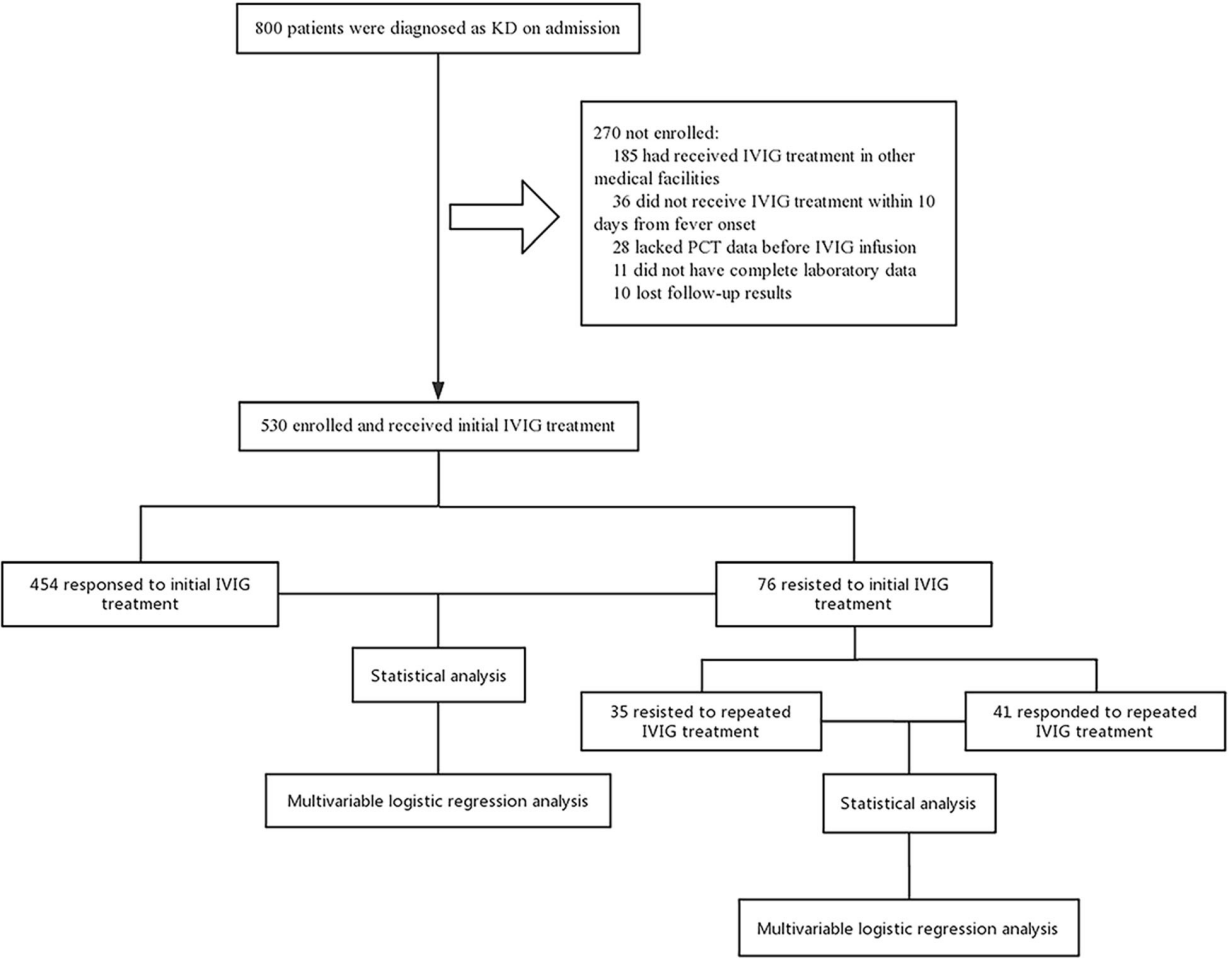
The flowchart of our prospective study. A total of 800 patients were diagnosed as KD on admission. Those who had received IVIG treatment at other medical facilities without serum PCT value (*n* = 185) or did not receive IVIG treatment within 10 days from fever onset (*n* = 36) were firstly excluded. Another 28 patients were excluded due to lack of PCT data before IVIG infusion. Additionally, we excluded 21 patients because of incomplete other laboratory data (*n* = 11) or lack of follow-up results (*n* = 10). Finally, the data of 530 patients were analyzed, including 76 initial IVIG-resistant patients and 454 IVIG-responsive patients. Initial IVIG-resistant group were then divided into repeated responsive subgroup (*n* = 41) and repeated resistant subgroup (*n* = 35). Then data analysis and multivariate logistic regression analysis were performed in these groups.

In total, 800 patients were diagnosed with KD on admission. Patients presented with other diseases such as infectious diseases, systemic autoimmune diseases, cardiogenic shock and major trauma were not included. Of the 800 KD patients, those who had received IVIG treatment at other medical facilities without serum PCT value (*n* = 185) or did not receive IVIG treatment within 10 days from fever onset (*n* = 36) were firstly excluded. Another 28 patients were excluded due to lack of PCT data before IVIG infusion. Additionally, we excluded 21 patients because of incomplete laboratory data (*n* = 11) or lack of follow-up results (*n* = 10). Finally, the data of 530 patients were analyzed (Fig. 1).

Serum samples were obtained to measure serum PCT levels using the electro-chemiluminescence immunoassay assay (ECLIA) on Roche Cobas e114 analyzer (Roche, Switzerland) on the day before IVIG was started. The analytical range of PCT concentration was 0.01-100 ng/ml. At the same time, other laboratory parameters were also obtained and analyzed. The subgroup division of PCT level was based on the validated thresholds of PCT for the management patients with infections and sepsis (< 0.05 ng/ml, 0.05–0.5 ng/ml, 0.5–2.0 ng/ml, 2.0–10.0 ng/ml and ≥ 10.0 ng/ml). Additionally, using a cut-off value of 0.5 ng/ml, the patients were categorized into normal and abnormal PCT groups.

All patients received the same standard treatment program of KD. High-dose IVIG (2 g/kg given as a single intravenous infusion) and aspirin (30–50 mg/kg/day) were administered within 10 days of illness onset. After patients defervesced, dose of aspirin decreased to 3–5 mg/kg/day and continued for 6–8 weeks. IVIG-resistance was defined as persistent or recurrent fever for > 36 h after completion of initial IVIG infusion. For patients with initial IVIG resistance, the second IVIG (2 g/kg given as a single intravenous infusion) was given according to the expert consensus for the diagnosis and treatment for KD in China. Furthermore, pulse intravenous methylprednisolone (10-30 mg/Kg/day for 3 consecutive days) followed by oral prednisone (2 mg/Kg/day) tapered for 7 days were applied as the additional treatment if the patient had recurrent or persistent fever even after the second IVIG administration.

CALs were defined on the normalization of dimensions for body surface area (BSA) as Z scores (standard deviation units from the mean, normalized for body surface area) as following: no involvement (z score < 2.0), dilation (z score ≥ 2.0 to < 2.5), aneurysm (z ≥ 2.5; z ≥ 10 for giant aneurysm) coronary arteries on the basis of the maximal internal diameters of the right coronary artery (RCA), left anterior descending artery (LAD) and left circumflex coronary artery (LCX). According to institutional protocol, patients underwent standardized echocardiograms by the same experienced pediatric ultrasonologists during the acute/subacute phase and 6 to 8 weeks later in cardiology clinic follow-up evaluations until the resolution of coronary artery abnormalities. BSA and z scores were calculated using the Haycock [29] and the Kobayashi equations [30], respectively.

## Statistical analysis

Data analysis was performed with SPSS 21.0 (SPSS Inc. Chicago, IL, USA). Quantitative data are presented as the median with the 25th and 75th percentiles (interquartile range (IQR)) in square brackets, while qualitative data are expressed as n/% as appropriate. Shapiro-Wilk test and homogeneity test of variance were used to confirm that quantitative data from different groups were normally distributed and meet the criteria for homogeneity of variance. The chi-square test and unpaired Student’s t test/Mann–Whitney U test were applied to compare the demographic characteristics, clinical manifestations and laboratory data between groups. Multivariable logistic regression analysis was applied to assess the association between serum PCT levels and IVIG resistance. Receiver operating characteristic (ROC) analysis was performed to determine the best cutoff value of serum PCT level and its validity for both initial and repeated IVIG resistance prediction. *P* values of < 0.05 were considered to be statistically significant.

## Results

### Subjects

There were 454 (85.7%) patients who responded to initial IVIG infusion and 76 (14.3%) children suffering from initial IVIG resistance. Of the 76 patients with initial IVIG resistance, 35 children did not respond to repeated IVIG treatment and received pulse intravenous methylprednisolone infusion. No patients received additional treatment such as infliximab, plasma exchange and cytotoxic agents. CALs were observed in 66 patients (12.5%), while transient pericardial effusion, valve regurgitation, cardiac enlargement and ventricular systolic dysfunction were noted in 13, 52, 53 and 3 children, respectively. A total of 191 patients (36.0%) were diagnosed as incomplete KD.

### Serum PCT level in KD patients

Of the 530 eligible KD patients, serum PCT levels in the acute phase before initial IVIG infusion ranged from 0.01 ng/ml to 79.03 ng/ml with a median of 0.72 ng/ml (IQR 0.23–2.14). The number of KD patients with serum PCT levels < 0.05 ng/ml, 0.05–0.5 ng/ml, 0.5–2.0 ng/ml, 2.0–10.0 ng/ml, and ≥ 10.0 ng/ml were 3, 222, 164, 114 and 27, respectively. No significant differences were found in serum PCT levels between CALs and non-CALs group (0.73[0.23–2.14]ng/ml vs 0.48[0.19–1.81]ng/ml, *p* = 0.841), as well as in patients with complete and incomplete KD (0.86[0.29–2.35]ng/ml vs 0.47[0.17–1.64]ng/ml, *p* = 0.969).

### Serum PCT level for initial IVIG resistance prediction

Table 1 summarized the comparison of the demographics, clinical characteristics, and laboratory values between the initial IVIG-responsive group and IVIG-resistant group. The mean age, fever duration prior to admission, sampling day of illness, day of illness before IVIG infusion, and the proportions of male sex, typical clinical features and incomplete KD between the two groups were not significantly different (all *p* > 0.05). Patients in the initial IVIG-resistant group presented with higher incidence of CALs (*p* < 0.001), pericardial effusion (*p* = 0.001), valve regurgitation (*p* = 0.021) and cardiac enlargement (*p* = 0.026) compared with those in IVIG-responsive group. In terms of the laboratory data, the nonresponders had a higher neutrophil-lymphocyte ratio (NLR) (*p* = 0.002), a higher creatinine (Cr) level (*p* = 0.009), a higher C-creative protein (CRP) level (*p* = 0.008), a lower platelet (PLT) count (*p* = 0.002), a lower albumin (ALB) concentration (*p* = 0.007), and a lower serum sodium (Na+) level (*p* < 0.001), Whereas no significant differences were found in any other laboratory variables including white blood cell, hemoglobin levels, erythrocyte sedimentation rate (ESR), alanine aminotransferase (ALT), aspartate aminotransferase (AST), total bilirubin (TB), urea nitrogen, potassium and cardiac troponin level (all *p* > 0.05).

**Table 1:** Comparison of clinical data between the groups of IVIG-resistant and IVIG-responsive in KD

		IVIG-responsive(*n* = 454)	*P* value
Age(months)	33.50[16.00–60.00]	24.00[13.00–42.00]	0.283
Male (%)	39(51.3%)	260(57.3%)	0.333
Day of illness before IVIG	5.00[5.00–6.00]	5.00[5.00–6.00]	0.199
Fever duration prior to admission, days	5.00[4.00–6.00]	5.00[4.00–6.00]	0.779
Sampling day of illness, days	5.00[3.25–5.75]	5.00[4.00–6.00]	0.240
Laboratory features			
WBC count(10^9^/L)	14.65[11.10–17.65]	13.45[10.88–16.80]	0.176^#^
NLR	4.78[2.76–9.37]	2.68[1.67–4.71]	0.002*^#^
Hemoglobin(g/L)	108[102.00–115.00]	108.00[101.00–116.00]	0.945
PLT count(10^9^/L)	295.50[234.00–346.00]	325.00[272.25–402.00]	0.002*
AST(IU/L)	37.50[24.25–68.75]	32.00[25.00–49.00]	0.337^#^
ALT(IU/L)	49.50[26.25–120.25]	36.00[21.00–77.00]	0.176
ALB(g/L)	36.00[31.05–39.00]	38.00[35.00–41.00]	0.007*
Total bilirubin(mg/L)	7.00[5.00–16.20]	6.00[4.00–8.00]	0.065
Cardiac troponin (ug/L)	0.01[0.01–0.02]	0.01[0.01–0.01]	0.214
Creatinine(umol/L)	29.00[26.25–37.75]	27.00[22.75–31.25]	0.009*^#^
Urea nitrogen(mmol/L)	2.90[2.34–3.81]	2.60[2.10–3.20]	0.208
Sodium(mmol/L)	134.00[132.70–137.00]	137.00[135.00–139.00]	< 0.001*
Potassium(mmol/L)	3.93[3.54–4.32]	4.14[3.77–4.50]	0.136
ESR(mm/h)	65.00[46.25–87.00]	64.50[47.75–82.00]	0.883^#^
CRP(mg/L)	89.50[58.25–144.00]	70.00[42.00–105.40]	0.008*
PCT(ng/ml)	1.70[0.47–5.61]	0.64[0.21–1.77]	0.009*^#^

The data are presented as the median with the 25th and 75th percentiles in square brackets for continuous variables and as the percentage for the categorical variables

Abbreviations: *WBC* white blood cell, *NLR* neutrophil-lymphocyte ratio, *PLT* platelet, *ESR* erythrocyte sedimentation rate, *CRP* C-reactive protein, *ALB* Albumin, *AST* aspartate aminotransferase, *ALT* alanine aminotransferase, *PCT* procalcitonin, *IVIG* intravenous immunoglobulin, *CALs* Coronary artery lesions, *KD* Kawasaki Disease

^#^Variables between two groups were compared by the Mann–Whitney U test due to abnormal data distribution

* Statistically significant (*P* < 0.05)

**Fig. 2:**
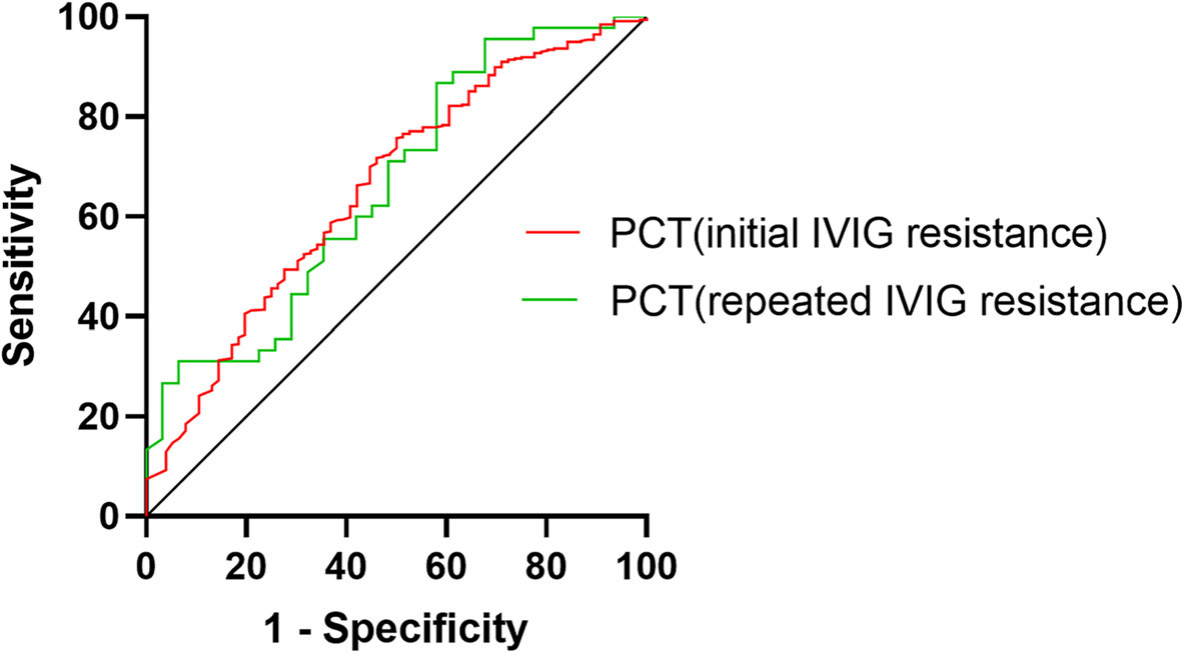
The receiver-operating characteristic (ROC) curve for PCT in predicting initial and repeated IVIG resistance

The serum PCT level was significantly higher in the initial IVIG-resistant group in comparison with the IVIG-responsive group (1.70[0.47–5.61]ng/ml vs 0.64[0.21–1.77] ng/ml, *p* = 0.009). However, after adjusted by NLR, PLT, ALB, Cr, Na + and CRP using the multivariate logistic regression analysis, serum PCT level was not identified as an independent factor for initial IVIG resistance (*P* = 0.986) (Table 2). The best PCT cutoff value for initial IVIG resistance prediction was 1.48 ng/ml, yielding a sensitivity of 53.9%, a specificity of 71.8%, a positive predictive value (PPV) of 24.3%, a negative predictive value (NPV) of 90.3% (Table 3). The area under the ROC curve was 0.658 (95% CI, 1.82–4.90, *p* < 0.001) (Fig. 2).

**Table2:** A multivariate logistic regression model for initial IVIG resistance in patients with KD

Variates	β	SE	Walds	*P* value	OR	95%CI
NLR	−0.072	0.028	6.445	0.011*	0.93	0.88–0.98
ALB	0.088	0.034	6.679	0.010*	1.09	1.02–1.17
PLT	0.003	0.001	4.184	0.041*	1.00	1.00–1.01
Cr	−0.002	0.008	0.071	0.790	1.00	0.98–1.01
CRP	0.003	0.003	1.020	0.312	1.00	1.00–1.01
Na+	0.080	0.039	4.235	0.040*	1.08	1.00–1.17
PCT	0.000	0.024	0.000	0.986	1.00	0.95–1.05

Abbreviations: *IVIG* intravenous immunoglobulin, *NLR* neutrophil-lymphocyte ratio, *ALB* Albumin, *PLT* platelet, *Na+* serum sodium, *PCT* procalcitonin, *Cr* Creatinine;

*Statistically significant (*P* < 0.05)

**Table 3:** The validity of PCT in predicting initial IVIG resistance for the total group and the abnormal PCT group

Initial IVIG resistance	Diagnostic test	Gold standard			Sen	Spe	PPV	NPV	Diagnostic accuracy	OR(95%CI)	P
Total group(*n* = 530)	PCT ≥ 1.48 ng/ml	positive	41	128	0.54	0.72	0.24	0.90	0.69	2.98 (1.82–4.90)	< 0.001*
		negative	35	326							
Abnormal PCT group(*n* = 305)	PCT ≥ 1.81 ng/ml	positive	38	110	0.67	0.56	0.26	0.88	0.58	2.51(1.37–4.60)	0.002*
		negative	19	138							

*CI* confidence ratio, *NPV* negative predictive value, *OR* odds ratio, *PCT* procalcitonin, *PPV* positive predictive value, *Sen* sensitivity;

*Spe* specificity;

*Statistically significant (*P* < 0.05)

The subgroup stratification analysis was further performed. For patients with normal PCT, no significant difference was evidenced in serum PCT levels between the initial IVIG responders and non-responders (0.21[0.15–0.34] ng/ml vs 0.20[0.12–0.31] ng/ml, *p* = 0.427). For patients with PCT > 0.5 ng/ml, there was borderline significant difference between two groups (2.90[1.25–6.00]ng/ml vs 1.56[0.91–3.80]ng/ml, *p* = 0.058). The best cutoff value of PCT for predicting initial IVIG resistance in abnormal PCT group was 1.81 ng/ml, yielding a sensitivity of 66.7%, a specificity of 55.6%, a PPV of 25.7%, a NPV of 87.9% and the diagnostic accuracy of 57.7% (Table 3).

### Serum PCT level for repeated IVIG resistance prediction

A total of 76 KD patients received repeated IVIG treatment. The comparison of the demographics, clinical characteristics, and laboratory values between repeated IVIG responders (*n* = 41) and nonresponders (*n* = 35) were illustrated in Table 4. Being similar with the groups of initial IVIG-responsive and IVIG-resistant, the mean age, fever duration prior to admission, sampling day of illness, day of illness before IVIG infusion, and the proportions of male sex, typical clinical features and incomplete KD were not significantly different in repeated IVIG-resistant and IVIG-responsive group (all *p* > 0.05). In terms of clinical outcomes, a higher proportion of CALs and cardiac abnormalities were noticed in repeated IVIG-resistant group compared with those in second IVIG-responsive group. However, besides pericardial effusion (*p* = 0.016) and CALs (*p* = 0.048), the followings (valve regurgitation, cardiac enlargement and ventricular systolic dysfunction) did not reach statistical significance (all *p* > 0.05). As for laboratory values, repeated nonresponders had a higher NLR (*p* = 0.033), a higher urea nitrogen (UN) level (*p* = 0.043), and a higher CRP level (*p* = 0.014). There was no significant difference in any other laboratory variables such as white blood cell, hemoglobin levels, PLT, ESR, ALT, AST, albumin (ALB), TB, Sodium, potassium, creatinine and cardiac troponin level (all *P* > 0.05).

**Table 4:** Comparison of clinical data between the groups of repeated IVIG-resistant and IVIG-responsive patients in KD

	IVIG-resistant(*n* = 35)	IVIG-responsive(*n* = 41)	*P* value
Age(months)	39.00[17.00–62.00]	32.00[15.50–48.40]	0.338
Male (%)	17(48.6)	22(53.7)	0.658
Day of illness before IVIG	5.00[4.00–6.00]	5.00[5.00–6.50]	0.343
Fever duration prior to admission, days	5.00[4.00–5.00]	5.00[4.00–6.50]	0.889
Sampling day of illness, days	4.00[4.00–5.00]	5.00[3.00–6.00]	0.065
Laboratory features			
WBC count(10^9^/L)	15.50[9.40–20.10]	14.60[11.25–16.35]	0.410
NLR	7.57[2.70–12.86]	3.97[2.80–7.29]	0.033*
Hemoglobin(g/L)	106.00[99.00–113.00]	111.00[102.00–117.50]	0.252
PLT count(10^9^/L)	290.00[233.00–342.00]	304.00[242.50–367.50]	0.347
AST(IU/L)	41.00[28.00–70.00]	32.00[23.00–73.00]	0.558^#^
ALT(IU/L)	48.00[34.00–118.00]	55.00[23.00–127.50]	0.974^#^
ALB(g/L)	34.00[29.00–39.00]	37.00[32.50–39.50]	0.201
Total bilirubin(mg/L)	7.00[4.70–30.00]	7.50[5.00–12.50]	0.084^#^
Cardiac troponin (ug/L)	0.01[0.01–0.02]	0.01[0.01–0.01]	0.930^#^
Creatinine(umol/L)	33.00[27.00–39.00]	29.00[24.50–37.00]	0.095
Urea nitrogen(mmol/L)	3.20[2.33–4.14]	2.90[2.35–3.40]	0.043*^#^
Sodium(mmol/L)	133.00[131.00–136.00]	135.00[133.00–137.50]	0.093
Potassium(mmol/L)	3.90[3.40–4.32]	3.94[3.61–4.28]	0.633
ESR(mm/h)	69.00[47.00–92.00]	64.00[43.50–80.50]	0.279
CRP(mg/L)	122.00[64.00–168.00]	76.00[50.50–112.50]	0.014*
PCT(ng/ml)	2.90[0.51–8.34]	1.43[0.37–3.37]	0.017*^#^

The data are presented as the mean ± SD for continuous variables and as the percentage for the categorical variables

Abbreviations: *WBC* white blood cell, *NLR* neutrophil-lymphocyte ratio, *PLT* platelet, *ESR* erythrocyte sedimentation rate, *CRP* C-reactive protein, *ALB* Albumin, *AST* aspartate aminotransferase, *ALT* alanine aminotransferase, *PCT* procalcitonin, *IVIG* intravenous immunoglobulin, *CALs* Coronary artery lesions, *KD* Kawasaki Disease

^#^Variables between two groups were compared by the Mann–Whitney U test due to abnormal data distribution

*Statistically significant (*P* < 0.05)

The repeated IVIG nonresponders presented with a remarkably higher serum PCT level compared to responders (2.90[0.51–8.34]ng/ml vs 1.43[0.37–3.37]ng/ml, *p* = 0.017). Similarly, after adjusted by NLR, UN and CRP, the multivariate logistic regression analysis failed to identify the serum PCT as an independent risk factor for repeated IVIG resistance prediction (*p* = 0.751) (Table 5). The discriminating cutoff value of PCT for repeated IVIG resistance prediction was 2.88 ng/ml, producing a sensitivity of 51.4%, a specificity of 73.2%, a PPV of 62.1% and a NPV of 63.8% (Table 6). The area under the curve was 0.620 (95%CI: 1.11–7.52, *p* = 0.028) (Fig. 2).

**Table 5:** A multivariate logistic regression model for repeated IVIG resistance in patients with KD

Variates	β	SE	Walds	*P* value	OR	95%CI
NLR	−0.031	0.044	0.499	0.480	0.97	0.89–1.06
UN	−0.168	0.224	0.565	0.452	0.85	0.55–1.31
CRP	−0.007	0.006	1.438	0.230	0.99	0.98–1.00
PCT	−0.021	0.067	0.100	0.751	0.98	0.86–1.12

Abbreviations: *IVIG* intravenous immunoglobulin, *NLR* neutrophil-lymphocyte ratio, *UN* Urea nitrogen, *PCT* procalcitonin

**Table 6:** The validity of PCT in predicting repeated IVIG resistance for the total group and the abnormal PCT group

Repeated IVIG resistance	Diagnostic test	Gold standard			Sen	Spe	PPV	NPV	Diagnostic accuracy	OR(95%CI)	P
Total group(*n* = 76)	PCT ≥ 2.88 ng/ml	positive	18	11	0.51	0.73	0.62	0.64	0.63	2.89 (1.11–7.52)	0.028*
		negative	17	30							
Abnormal PCT group(*n* = 57)	PCT ≥ 5.80 ng/ml	positive	12	4	0.44	0.87	0.75	0.63	0.67	5.20(1.42–19.04)	0.009*
		negative	15	26							

*CI* confidence ratio, *NPV* negative predictive value, *OR* odds ratio, *PCT* procalcitonin, *PPV* positive predictive value, *Sen* sensitivity;

*Spe* specificity;

*Statistically significant (*P* < 0.05)

For patients with normal PCT, no significant difference was found in PCT level between repeated IVIG responders and non-responders (0.18[0.14–0.32]ng/ml vs 0.22[0.19–0.41]ng/ml, *p* = 0.332). For patients with PCT > 0.5 ng/ml, the PCT level was significantly higher in the IVIG nonresponders than the IVIG responders (5.67 [1.58–11.56] ng/ml vs 2.09[1.05–4.74] ng/ml, *p* = 0.014), while the best cutoff PCT value for repeated IVIG resistance prediction was 5.8 ng/ml. However, the predictive value did not enhance with a lower sensitivity of 44.4%, despite the specificity was slightly elevated (Table 6).

## Discussion

In the present study, we prospectively explored the predictive value of serum PCT level for initial IVIG resistance in KD with the largest sample size. Most importantly, to the best of our knowledge, this was the first study to determine the validity of PCT in repeated IVIG resistance prediction. Furthermore, not only the sensitivity and specificity, but also the PPV and NPV were also assessed. It was revealed that serum PCT level was significantly elevated both in initial and repeated IVIG-resistance group in comparison with that in nonresponders. However, PCT may not be suitable as a single marker to accurately predict both initial and repeated IVIG resistance in a clinical setting because of its low sensitivities.

The definite cause of KD is currently unknown, it is however generally accepted that KD develops as a result of a genetic predisposition combined with an infection with an undefined trigger or an autoimmune mechanism [28], and always associated with elevated levels of inflammatory cytokines such as TNF-α and IL-6 [31, 32], which could in turn modulate the production and secretion of PCT [33]. Only a small number of patients in our cohort (*n* = 3) presented with PCT levels below 0.05 ng/ml, and the majority had very elevated levels (2.0–10.0 ng/ml in 114 cases and > 10.0 ng/ml in 27 cases). These data suggested that PCT might be useful in differentiating KD from viral infections and autoimmune diseases that present in a clinically similar way. Indeed, this observation was in agreement with previous studies [25, 28].

Accumulating evidences have found the inflammatory cytokines such as TNF-α and IL-6 would excessively release in the acute phase of KD [31, 32, 34]. The cytokine profile may reflect the disease severity and is associated with the development of IVIG resistance, suggesting the potential role of serum PCT level in IVIG resistance prediction. Currently, the predictive value of serum PCT level for initial IVIG resistance in KD is limited. In the study conducted by Dominguez et al. [26], it was found that KD patients with a PCT ≥ 0.5 ng/ml had a significantly higher incidence of IVIG resistance (29% vs 7%, *P* = 0.02). Another study from Korea [28] documented a lower proportion of IVIG nonresponders than responders had PCT levels< 0.25 ng/ml. However, both of studies were limited by small sample size (*n* = 85 and 49, respectively) and retrospective nature. Additionally, the discriminating cut-off value of PCT was not determined and the corresponding predictive value was unknown. Furthermore, multivariate logistic regression analysis was also not performed. A larger study [27] measuring serum PCT concentration in 160 Japanese KD patients showed serum PCT was significantly higher in nonresponders to an initial IVIG therapy than in responders. A cutoff value 0.5 ng/ml for nonresponders produced a sensitivity of 85.0% and a specificity of 64.0%. However, unlike previous studies, we found the validity and clinical application of PCT as a single biomarker for initial IVIG resistance prediction should be cautious due to a relatively low sensitivity of 0.54 and PPV of 0.24. In addition, a further multivariate logistic regression analysis failed to identify serum PCT level as an independent risk factor for initial IVIG resistance in KD. This finding may be explained by a correlation between PCT and other variables incorporated into the regression model. Alternatively, PCT may be less sensitive as a single biomarker for initial IVIG resistance prediction compared to conventional parameters such as NLR [35, 36], ALB [19, 37, 38], PLT [16, 17, 20] and Na+ [16, 22]. Given a sufficient number of patients and prospective approach, the findings in our report may be more conclusive.

In terms of the repeated IVIG resistance prediction in KD, paucity of data was currently available and the role of serum PCT in this issue has never been investigated. Despite several clinical trials from Japan documented addition of corticosteroid [39, 40] or ciclosporin therapy [41] to standard-dose IVIG and aspirin in the primary therapy of KD reduced the initial non-response rate and decrease the incidence of CALs among high-risk patients for initial IVIG resistance predicted by Kobayashi [16], Sano [18], and Egami [42] scores, the non-response rate still remained approximately 10–20% [43]. These findings suggested that high-risk KD patients for IVIG resistance might mostly benefit from aggressive therapy and prediction of repeated IVIG resistance was equally essential and clinically significant. In the present study, we firstly found the repeated IVIG nonresponders presented with a remarkably higher serum PCT level compared to responders. A cutoff value 2.88 ng/ml for repeated nonresponders yielded a relatively moderate sensitivity of 51.4%, specificity of 73.2%, PPV of 62.1% and NPV of 63.8%. Obviously, we could not identify all the non-responders for repeated IVIG by detecting serum PCT level, these data, however, may expand the limited information regarding repeated IVIG resistance prediction and provide some references for clinical management.

Additionally, the association between serum PCT level and development of CALs were also investigated in this report. The first study [25] exploring the use of PCT as a predictive tool for CALs in KD children found that a PCT > 3.0 ng/ml was correlated with coronary aneurysm development in affected patients. This study was limited by a small sample size of 25 patients and non-standard IVIG treatment regimen (400 mg/kg for 5 days). Another study conducted by Catalano-Pons et al. [44] demonstrated that no significant difference was found in the value of PCT between patients with or without coronary aneurysms. Being similar with the first study, this study was also limited by small sample (*n* = 18). Two subsequent researches from Korea [28] and US [26] did not support the use of PCT as a predictor for the development of CALs. Our study hence agreed with the findings of the latter ones that no significant correlation between the presence of CALs and PCT was evident. These collective evidences may indicate serum PCT was not suitable for CALs prediction.

This study must be viewed in light of some potential limitations. First, this study was performed in a single institution and because our hospital is the largest Children Medical Center in Southwest China, it might therefore lead to some selective bias that more severe patients being admitted to our hospital. Second, the present study was a prospective cohort study and had strict inclusion and exclusion criteria. The findings in our study were only applicable to KD patients receiving the standardized IVIG treatment within 10 days from fever onset.

Despite above limitations, this study is the first to determine the predictive value of serum PCT for both initial and repeated IVIG resistance with a sufficient number of patients and prospective approach. We found serum PCT levels were significantly higher in IVIG nonresponders, but it may only serve as a complementary laboratory marker for the prediction of both initial and repeated IVIG resistance prediction in KD. Given the unknown origin of KD, we suggest that a prediction model combining other specific indicators rather than clinical and routine laboratory variables might have a better performance.

## Conclusion

Serum PCT level was significantly higher in IVIG nonresponders, but it may not be suitable as a single marker to accurately predict both initial and repeated IVIG resistance in KD.

## Data Availability

All data are included in this published article.

## Abbreviations

ALB: Albumin
AUC: Area under the curve
CALs: Coronary artery lesions
CI: Confidence interval
CRP: C-reactive protein
IL: Interleukin
IVIG: Intravenous immunoglobulin
KD: Kawasaki disease
Na+: Serum sodium
NLR: Neutrophil-lymphocyte ratio
NPV: Negative predictive value
PCT: Procalcitonin
PLT: Platelet
PPV: Positive predictive value
ROC: Receiver operating characteristic
TNF: Tumor necrosis
UN: Urea nitrogen
